# Ruptured Sylvian arachnoid cysts: an update on a real problem

**DOI:** 10.1007/s00381-022-05685-3

**Published:** 2022-09-28

**Authors:** L. Massimi, F. Bianchi, A. Benato, P. Frassanito, G. Tamburrini

**Affiliations:** 1 Pediatric Neurosurgery, Fondazione Policlinico Universitario, A. Gemelli IRCCS, Rome, Italy; 2 Catholic University of the Sacred Heart, Rome, Italy

**Keywords:** Arachnoid cysts, Sylvian, Rupture, Neuroendoscopy, Microsurgery, Subdural collection, Hygroma

## Abstract

**Purpose:**

Sylvian arachnoid cysts (SACs) are the most common type of arachnoid cysts and the most prone to undergo a rupture. This event is considered rare but potentially severe. No definite information is available on its occurrence or management. The goal of the present article is to provide an update on the epidemiological, etiological, and clinical aspects and the management of this peculiar clinical condition.

**Methods:**

A comprehensive review of the English literature of the last 40 years on this topic has been realized. Moreover, a personal series of children investigated and treated in the last 20 years is presented. These patients were managed as follows: (1) treatment of the subdural collection; (2) identification of candidates for surgical treatment of the residual cyst (brain MRI, perfusion brain MRI, prolonged invasive ICP monitoring (selected cases), EEG, neuropsychological tests); (3) surgical treatment of the cyst in the patients with pathological perfusion MRI and/or ICP measurement and/or clear neurophysiological and neuropsychological correlations.

**Results:**

A total of 446 patients (430 from the literature and 16 from the personal series), mainly children, adolescents, and young adults, have been analyzed leading to the following results: (1) SAC rupture is rare but not negligible (yearly risk of rupture: 0.04%; overall risk up to 10% in children affected by SCAs). Prophylactic surgery in asymptomatic cases is not advisable. (2) The mechanism of rupture is not known but an impact of SAC against the sphenoid wing and/or a direct injury on SAC through a thinned temporal bone, with possible laceration of the cyst wall vessels and/or tear of the bridging veins, can be hypothesized. A head injury is often not reported (may be misdiagnosed). (3) Subdural collection (hygroma > chronic hematoma) is the most common finding followed by intracystic bleeding, extradural hematoma, and other types of bleeding. Signs or symptoms of raised intracranial pressure are the most frequent ones. (4) The complication of the rupture is usually treated in emergency or in the acute period by burr hole or craniotomic evacuation of the subdural collection, although a conservative management is possible in some cases. Following the rupture, the majority of SACs are treated (70%), often at the same time of the complication, but no specific investigations are routinely performed to select candidates. According to our protocol, only 43.7% of SACs needed to be treated.

**Conclusions:**

The “spontaneous” or posttraumatic rupture of SACs is a rare but potentially significant complication followed by a generally good outcome. The course of the cyst is independent from the outcome of the complication, consequently requiring specific investigations for individuating those lesions interfering with CSF dynamics and/or cerebral blood flow.

## Introduction

Sylvian arachnoid cysts (SACs) are congenital lesions accounting for 0.1–0.3% of all intracranial masses in autoptic series and up to 0.5–1% in clinical and radiological series [1]. Their prevalence among the population is not negligible, being estimated to be slightly lower than 2% [2, 3]. SACs, which represent about 50% of all arachnoid cysts in the general population (up to 75% in children), are often diagnosed as incidental findings, remaining asymptomatic during the life in most of the cases [4].

The major complication occurring in SACs, which can make them quickly symptomatic, is represented by their post-traumatic or “spontaneous” rupture, usually presenting as subdural fluid collection or intra-cystic hemorrhage [5]. This event seems to be peculiar of SACs where the risk of rupture is higher compared with intracranial arachnoid cysts located elsewhere [6, 7]. The cyst rupture raises a great interest among the scientific community because of the possible emergency implications and the variable clinical presentation and course, as demonstrated by the large number of reports published even in recent years [8–16]. Moreover, the management of ruptured SACs still represents a controversial problem for neurosurgeons as far as the surgical indication and the type of treatment are concerned. This controversy is particularly felt in the pediatric population where, being temporal fossa cysts often asymptomatic, their rupture is the most common indication for surgery [17].

The goal of the present article is to review the pertinent literature on this topic to provide an update on the epidemiological, etiological, and clinical aspects and the management of this clinical condition. The authors’ personal series and management protocol for ruptured SACs in children are briefly reported, too.

## Materials and methods

### Review of the literature

The authors performed a comprehensive review of the English literature on the matter through PubMed, Embase, Scopus, and Cochrane library. The research strings were “arachnoid cyst,” “Sylvian arachnoid cyst,” “middle temporal fossa arachnoid cysts,” and “temporal arachnoid cyst.” The aforementioned strings were searched as standalone sentence and coupled with the word “rupture” and “trauma” or “spontaneous.” All papers reporting enough information on ruptured SCAs were reviewed, including case reports, namely for epidemiological and clinical purposes.

### Personal series

All children consecutively admitted to our institution for ruptured SACs in the last 20 years (2000–2019, minimum follow-up: 2 years) were considered for this retrospective analysis. Patients without complete clinical, radiological, and outcome data were excluded.

The management protocol can be summarized as follows: (1) Management of the rupture in emergency with brain CT scan (and, if possible, MRI) and surgery. A burr hole approach is used to evacuate the subdural fluid collection and a transient external drainage is left in place for at most 7 postoperative days, waiting for the resolution of the collection. A permanent subduro-peritoneal shunt is placed only in case of persistent hygroma and removed thereafter (once the hygroma is disappeared); (2) investigations to identify possible candidates to the surgical treatment of the residual cyst. These examinations are started about 6 months after the treatment of the complication and consist of (a) brain MRI, to verify the resolution of the complication and possible changes of the cyst (disappearance, increase in size, etc.); (b) perfusion brain MRI (brain 99mTC-Spect in the first 10 years), to detect possible cerebral hypoperfusion around the cyst as sign of brain suffering; (c) prolonged invasive ICP monitoring (24–72 h), to look for possible intracranial hypertension, only in selected cases (patients with persistence of the cyst and signs/symptoms of raised ICP); (d) serial EEG checking for possible cyst-related anomalies; (e) neuropsychological tests seeking for possible associated cognitive or behavioral correlations. (3) Surgical treatment of the cyst in the patients with pathological perfusion MRI and/or ICP measurement and/or clear neurophysiological and neuropsychological correlations. Neuro-endoscopic approach is the first choice followed by microsurgery (cysto-peritoneal shunt only in case of repeated failure of the previous two approaches); (4) serial clinical and MRI assessments (timing according to the patients’ age) in both operated on and not operated on cases.

## Results

### Review of the literature

Overall, 430 cases of ruptured SACs have been found within 124 papers in the reviewed literature. The majority of these cases were described as isolated cases or small series, apart from 3 series of 32 [18], 44 [19], and 60 cases [20], respectively. The main characteristics of these patients are reported in detail in Table 1 [6–8, 10–16, 18–129]. The results are summarized in Table 2 and analyzed in the “Discussion” section.

**Table 1:** Review of the literature

**Paper**	**Number of cases**	**Age (years)/sex**	**Lateralization/Galassi classification**	**Trauma phenomenology**	**Symptoms**	**Complication**	**Surgical evacuation**	**Cyst treatment**	**Follow-up**
Abbas [21]	1	25/M	B/II	Street accident	LOC	EDH	No	None	Complete recovery
Adin [22]	2	36/M	U (right)/I	None	Headache, vomiting	CSDH	Craniotomy	None	Complete recovery
		21/M	U (left)/I	None	Headache	CSDH	No	None	
Albuquerque [23]	4	25/M	B/-	Assault	LOC	SDH	Burr holes	None	-
		9/-	U/II	Head trauma	LOC	SDH	Burr holes	None	
		25/-	IU/I	Head trauma	LOC	SDH	Burr holes	SDP shunt	
		10/-	U/II	None	Headache	SDH	Burr holes	None	
Amelot [19]	44	-	19—I 14—II 11—III	-	Headache, intracranial hypertension	-	6 burr hole 18 SDP shunt	3 microsurgery 1 endoscopy 9 CP shunt 7 none	37 improved
Auer [24]	5	31 mean	U/II	Head trauma	-	CSDH	-	-	-
Aydogmus [25]	1	15/M	U (left)/II	None	Headache	ASDH	Craniotomy	Craniotomy	Complete recovery
Balestrino [26]	17	8,3 mean	2B, 15U 1—I 9—II 7—III	None	Headache	6 CSDH, 11 SDH	1 burr hole 1 SDP shunt	15 endoscopy	16 complete recovery 1 partial recovery
Beretta [27]	1	20 s	U (left)/I	Sport accident	Headache, vomiting	CSDH	Burr hole	None	Complete recovery
Bilginer [28]	3	15/M	U (left)/II	None	Headache	CSDH	Burr hole	None	Complete recovery
		28/F	U (left)/II	Cesarean section with epidural anesthesia	Headache, vomiting	CSDH	Burr hole	None	Complete recovery
		12/M	U (left)/II	None	Headache, vomiting	CSDH + IH	Burr hole	None	Complete recovery
Bora [8]	1	2/M	U (left)/II	None	None	CSDH	No	None	Stable condition
Bovitsias [29]	1	56/M	U (left)/II	Head trauma	LOC	EDH	Craniotomy	None	Complete recovery
Bristol [30]	1	17/M	U (right)/II	Sport accident	Headache	SDH	Craniotomy	Microsurgery	Complete recovery
Burken [31]	1	39/F	U (right)/I	Head trauma	Headache	ASDH + IH	Craniotomy	Microsurgery	Partial recovery
Canty [32]	1	0/F	B/II	Head trauma	LOC	ASDH	Medical treatment	None	Complete recovery
Cappelen [33]	1	17/M	U (right)/II	Head trauma	Papilledema	CSDH	Craniotomy	Microsurgery	Complete recovery
Cayli [34]	1	12/F	U (left)/III	None	Headache	SDH	Craniotomy	Microsurgery	Complete recovery
Chan [35]	1	29/M	U (left)/II	Street accident	Headache	CSDH	Burr hole	None	Complete recovery
Chan [36]	3	2/-	U (left)/-	None	-	CSDH	Burr hole + SDP shunt	None	-
		10/-	U (right)/-	Head trauma	-	SDH	SDP shunt	None	-
		8/-	U (left)/-	None	Headache	SDH	SDP shunt	Microsurgery + CP shunt	-
Chandra [37]	1	12/M	U (left)/II	None	Headache, vomiting, LOC	CSDH + IC	Craniotomy	Microsurgery	Complete recovery
Chillala [38]	1	21/M	U (left)/III	Sport accident	Headache	CSDH	-	-	Complete recovery
Choong [39]	1	9/F	U (left)/II	None	Headache, vomiting, papilledema	SDH	Medical treatment (Diamox)	None	Complete recovery
Cress [6]	12	6–10	-	-	-	-	-	-	-
Cullis [40]	1	11/M	U (left)/II	None	Headache	CSDH	Craniotomy	Microsurgery	-
Darmoul [41]	1	25/M	-	Head trauma	Headache, vomiting	EH	Medical treatment	-	Complete recovery
De [42]	1	2/M	U (left)/II	Head trauma	Headache	CSDH	Craniotomy	None	Complete recovery
Demetriades [44]	1	24/M	U (left)/II	Sport accident	Headache, nausea	CSDH	Burr hole	None	Complete recovery
De Recondo [43]	1	47/F	U (left)/III	Head trauma	Headache	CSDH + IH	No	None	Complete recovery
Di Gaeta [45]	1	76/M	U (left)/III	None	Mild headache, paresthesia, visual deficit	CSDH	Craniotomy	Microsurgery	Complete recovery
Domenicucci [46]	5	38.3 mean	2 U (left)/II 3 U (right)/II	1 sport accident 1 assault 3 Street accident	-	CSDH	Burr hole	None	Complete recovery
Donaldson [47]	2	14/M	U (left)/II	Sport accident	Headache, vomiting, papilledema	SDH	Craniotomy	Microsurgery	Complete recovery
		5/M	U (right)/II	Head trauma	Headache, vomiting, papilledema	SDH	Craniotomy	Microsurgery	Complete recovery
Edmondson [48]	1	14/M	-	Sport accident	Headache	CSDH	Craniotomy	Microsurgery	Complete recovery
Ergun [49]	1	14/M	U (left)/III	None	Headache, vomiting, hemiparesis	SDH	Craniotomy	Microsurgery	Complete recovery
Eustace [50]	1	11/F	U (left)/III	None	Headache	CSDH + IH	Craniotomy	Microsurgery	Complete recovery
Furtado [10]	1	8/M	U (left)/III	Sport accident	Headache	IH	Craniotomy	Microsurgery	Complete recovery
Galarza [51]	2	17/M	U (left)/III	None	Headache, papilledema	CSDH	Craniotomy	Microsurgery	Complete recovery
		12/M	U (left)/III	Head trauma	Headache	CSDH + IH	Craniotomy	Microsurgery	Complete recovery
Galassi [53]	1	-	U (left)/II	Head trauma	-	CSDH	Craniotomy	Microsurgery	Complete recovery
Galassi [54]	2	13/M	U (left)/II	Street accident	LOC	EDH	Craniotomy	Microsurgery	Complete recovery
		16/M	U (left)/III	Street accident	LOC	EDH	Craniotomy	Microsurgery	Complete recovery
Galassi [52]	13	-	-	-	-	5 CSDH 4 SDH 2 IH 2 EDH	Craniotomy	Microsurgery	-
Gelabert-Gonzalez [55]	3	13/M	U (left)/II	Sport accident	Headache, vomiting	SDH	Craniotomy	Microsurgery	Complete recovery
		12/M	U (left)/III	Head trauma	Headache, seizure	SDH	Craniotomy	Microsurgery	Complete recovery
		6/M	U (left)/III	None	Headache, vomiting, LOC	SDH	Burr hole + SDP shunt	None	-
Gil-Gouveia [56]	1	16/F	U (left)/I	None	Headache	CSDH	Burr hole + SDP shunt	None	Complete recovery
Gunduz [57]	2	57/M	U (left)/III	None	Headache, TIA	IH	Craniotomy	Microsurgery	Complete recovery
		19/F	U (left)/III	None	Headache	CSDH + IH	Craniotomy	Microsurgery	Complete recovery
Gupta [58]	1	22/M	U (left)/II	Sport accident	Headache	SDH	Burr hole	None	Complete recovery
Hagan [59]	1	1/M	U (left)/III	Head truama	LOC	CSDH	None	None	Complete recovery
Hall [60]	1	34/M	U (right)/II	None	Headache, vomiting	CSDH	Craniotomy	Microsurgery	Complete recovery
Hara [62]	1	13/M	U (right)	-	-	CSDH	-	-	-
Hamada [61]	1	15/M	U (left)/II	Sport accident	Headache, vomiting, papilledema	CSDH	Craniotomy	Endoscopy	Complete recovery
Hamidi [11]	2	12/M	U (right)/II	Sport accedent	Headache	SDH	Medical treatment	None	Complete recovery
		15/M	U (left)/III	Head trauma	Headache, vomiting	SDH	-	-	-
Hasegawa [63]	1	5/M	U (left)/II	Head trauma	Headache, vomiting	CSDH	Burr hole	Craniotomy (delayed)	Complete recovery
Henriques [64]	1	10/M	U (left)/II	None	Headache, vomiting, papilledema	SDH	No	None	Complete recovery
Hong [65]	1	11/F	U (left)/III	None	Headache, vomiting	CSDH + IH	Craniotomy	Microsurgery	Complete recovery
Hopkin [66]	1	12/M	U (right)/III	Head trauma	Headache, papilledema	CSDH	SDP shunt	CP shunt	Complete recovery
Iaconetta [67]	1	13/M	U (right)/III	None	Headache, vomiting, papilledema	CSDH + IH	Craniotomy	Microsurgery	Complete recovery
Ibarra [68]	1	11/M	U (left)/II	None	Headache, vomiting, VI palsy	CSDH + IH	Burr hole	None	Complete recovery
Ildan [69]	1	32/M	U (right)/III	Head trauma	Headache, vomiting, papilledema	IH	Craniotomy	Microsurgery	Complete recovery
Inoue [70]	1	7/M	U (right)	None	Vomiting	CSDH	No	None	Complete recovery
Isik [71]	1	13/M	U (left)	Sport accident	Headache, vomiting	CSDH	Burr hole	None	Complete recovery
Kadiogly [72]	1	37/M	U (right)	Assault	Hemiparesis	EDH	Craniotomy	Microsurgery	-
Kaszuba [73]	1	47/M	U (left)/III	None	Headache, vomiting	CSDH + IH	Craniotomy	Microsurgery	Complete recovery
Katsaros [74]	1	35/F	U (right)/III	None	Headache	IH	No	None	Complete recovery
Kawanishi [75]	2	14/M	U (left)/II	Sport accident	Headache, nausea	CSDH	Burr hole	Craniotomy (delayed)	Complete recovery
		11/M	U (left)/II	Sport accident	Headache, vomiting	CSDH + IH	Burr hole	None	Complete recovery
Kertmen [76]	1	12/M	U (left)/II	Sport accident	Headache	CSDH	Burr hole	None	Complete recovery
Khilji [12]	1	9/M	U (left)/II	None	Headache, vomiting	SDH	Burr hole + SDP shunt	Microsurgery	Complete recovery
Kieu [13]	1	33/F	U (left)/II	None	Headache	CSDH	Craniotomy	Microsurgery	Complete recovery
Kim [77]	2	11/M	U (right)/III	Head trauma	Headache, vomiting	CSDH	Burr hole	None	Complete recovery
Kulali [78]	2	15/M	U (right)	None	-	EDH	Craniotomy	Microsurgery	Complete recovery
		6/M	U (right)	Street accident	-	EDH	Craniotomy + SDP	Microsurgery	Complete recovery
LaCour [79]	3	16/F	U (left)/II	None	Headache, vomiting, hemiparesis	ASDH	Craniotomy	Microsurgery	-
		31/M	U (left)/III	None	Headache	CSDH	Craniotomy	Microsurgery	-
		64/F	U (left)/III	None	Confusion, apathy	CSDH	Craniotomy	Microsurgery	-
Li [14]	1	14/M	B/III	None	Headache	CSDH	None	None	Complete recovery
Lipinski [80]	1	1.5/F	U (left)/III	Head trauma	Decreased vigilance, seizures	ASHD	Burr hole	None	Complete Clinical recovery
Liu [82]	3	10/M	U (left)/II	Head trauma	Headache	SDH	Craniotomy	Microsurgery	Complete Clinical recovery
		5/F	U (right)/II	None	Headache, irritability	SDH	Craniotomy	Microsurgery	Complete Clinical recovery
		2/M	U (left)/II	None	Developmental delay, seizures	SDH	Craniotomy	Microsurgery	Improvement in developmental milestones
Liu [81]	1	3/M	U (left)/III	None	None	None	None	None	Spontaneous disappearance of the cyst
Liu [15]	1	7/M	U (left)/III	None	Headache, vomiting	CSDH	Burr hole	Endoscopy	Complete recovery
Lohani [83]	1	11/M	U (right)/I	None	Headache, refractory back and leg pain	Intracranial and spinal SDH	None	None	Spontaneous resolution of hematoma, cyst reduction
Maeda [84]	1	13/M	U (left)/I	Head trauma	Headache	SDH	Craniotomy	Microsurgery	Complete Clinical recovery, cyst reduction
Maher [85]	8	10/M	U (left)/III	Head trauma	Headache, papilledema, VI CN palsy	Unilateral subdural hygroma	None	None	Clinical recovery; hygroma resolution, cyst persistence
		12/F	U (right)	Head trauma	Headache, nausea, vomiting	Unilateral subdural hygroma	None	None	Clinical recovery; hygroma resolution, cyst persistence
		16/M	U (right)/III	Head trauma	Transient headache	Bilateral subdural hygromas	None	None	Hygroma and cyst reduction
		8/M	U (right)	Head trauma	Headache	Bilateral subdural hygromas	None	None	Hygroma resolution, cyst persistence
		1/F	U (left)	None	Abnormal increase in head circumference	Unilateral subdural hygroma	None	None	Hygroma resolution, cyst persistence; normal development
		1/M	U (left)	None	Vomiting, increase in head circumference	Bilateral subdural hygromas	None	None	Hygroma and cyst reduction; normal development
		10/M	U (right)/II	Head trauma	Headache, nausea, vomiting	Bilateral subdural hygromas	None	None	Hygroma and cyst reduction
		7/M	U (right)	None	Headache, papilledema	Unilateral subdural hygroma	Craniotomy	Microsurgery, cystoperitoneal shunt	Clinical recovery; hygroma resolution, cyst reduction
Mao [18]	32	-	-	-	-	CSDH	-	-	-
Marnat [86]	1	16/M	-	-	-	CSDH	Embolization	-	Complete recovery
Marques [87]	1	29/M	U (left)/II	Physical exertion	Headache, vomiting, papilledema	Bilateral subdural hygromas	Craniotomy	Microsurgery	Clinical recovery; hygroma and cyst resolution
Mastronardi [88]	1	15/M	U (left)/II	Head trauma	Coma, anisocoria	ASDH	Craniotomy	None	Clinical recovery; cyst persistence
McDonald [89]	1	2/M	U (left)/II	None	Increased head circumference	None	None	None	Complete spontaneous cyst regression
Meshkini [91]	11	0–2	-	-	-	9 IH 2CSDH	-	-	-
Molloy [92]	1	18/M	U (right)/III	Assault	Headache, vomiting	EDH	No	VP shunt	-
Mori [7]	11	11/F	U (right)/I	None	Headache, vomiting	CSDH	Burr hole	None	Clinical recovery
		17/M	U (right)/I	Head trauma	Headache, vomiting	CSDH	Burr hole	None	Clinical recovery
		9/F	U (right)/II	Head trauma	Headache, vomiting	CSDH	Burr hole	None	Clinical recovery
		39/F	U (left)/II	Head trauma	Hemiparesis	CSDH	Burr hole	None	Clinical recovery
		12/M	U (right)/I	Surgery (cyst fenestration)	None	CSDH	Burr hole	Microsurgery	Clinical recovery
		41/M	U (left)/I	Head trauma	Headache, vomiting	CSDH	Burr hole	None	Clinical recovery
		5/M	U (left)/I	Head trauma	Headache	CSDH	Burr hole	None	Clinical recovery
		71/M	U (left)/I	None	Headache, gait disturbance, dementia	CSDH	Burr hole	None	Clinical recovery
		43/F	U (left)/I	Head trauma	Headache, hemiparesis	CSDH	Burr hole	None	Clinical recovery
		14/M	U (left)/I	Head trauma	Headache, hemiparesis	CSDH	Burr hole	None	Clinical recovery
		39/M	U (left)/I	Head trauma	Headache	CSDH	Burr hole	None	Clinical recovery
Nadi [93]	1	7/M	U (left)/III	Head trauma	Headache	ASDH	None	None	Hematoma resolution, cyst reduction
Ochi [94]	7	9/M	U (left)/II	NR	NR	SDH	NR	NR	NR
		31/M	U (left)	NR	NR	SDH	NR	NR	NR
		26/M	U (right)/III	NR	NR	SDH	NR	NR	NR
		16/M	U (left)	NR	NR	SDH	NR	NR	NR
		18/F	U (right)	NR	NR	SDH	NR	NR	NR
		18/M	U (right)	NR	NR	SDH	NR	NR	NR
		33/M	U (right)	NR	NR	SDH	NR	NR	NR
Offiah [95]	1	8/M	B/II	Head trauma	Headache, vomiting, double vision	Right subdural hygroma	Burr hole; subdural-peritoneal shunt	None	Hygroma resolution; increase in right cyst size
Oka [96]	3	17/M	U (right)/III	Head trauma	Headache	SDH	Burr holes	None	Hematoma resolution, cyst reduction
		24/M	U (left)/III	None	Headache	SDH	Burr holes	None	Hematoma resolution, cyst reduction
		13/M	U (left)/II	Head trauma	Headache, hemiparesis	SDH	Burr holes	None	Hematoma resolution, cyst reduction
Oliver [97]	1	21/M	U (right)	Head trauma	Headache, vomiting, papilledema, III CN palsy	SDH	Craniotomy	Microsurgery	Clinical recovery
Page [98]	7	15/M	U (right)/II	Head trauma	Headache, vomiting, papilledema	CSDH	Craniotomy	Microsurgery	Cyst and hematoma resolution
		17/M	U (right)/III	None	Headache, vomiting	CSDH	Craniotomy	Microsurgery	Clinical recovery
		11/M	U (left)/III	Head trauma	Headache, vomiting, papilledema, double vision	CSDH	Burr holes; subdural-peritoneal shunt; shunt removal, craniectomy, cranioplasty	None	Clinical recovery
		23/F	U (right)/II	Head trauma	Headache, vomiting, papilledema	CSDH	Burr holes	Cysto-peritoneal shunt	Hematoma resolution, cyst reduction
		57/M	U (left)/II	Head trauma	Headache, vomiting	CSDH	Craniotomy	Microsurgery	Hematoma resolution, cyst reduction
		17/M	U (left)/II	None	Headache, papilledema, photophobia	CSDH	Burr holes	None	Hematoma resolution, cyst reduction
		11/F	U (left)/II	None	Headache, vomiting, papilledema	CSDH	Craniotomy	Microsurgery	Clinical recovery
Paik [99]	1	19/M	U (left)/III	Head trauma	Headache	CSDH	Burr holes	None	Hematoma resolution, cyst reduction
Parsch [100]	16	5–80	U (left 56%; right 44%)	Head trauma (87%)	Symptoms of raised ICP (31%), hemiparesis (25%), seizures (6%)	Hygroma (75%), CSDH (25%)	Conservative (12%); craniotomy (12%); burr holes (62%), external subdural drain (12%)	Microsurgery (6%)	Clinical recovery (75%); minor symptoms (18%); 1 death due to cardiorespiratory complications
Pascoe [101]	1	43/M	U (left)/III	Head trauma	Headache, diplopia	CSDH	Craniotomy	Microsurgery	Clinical recovery
Patel [103]	1	22/M	U (left)/II	None	Headache, vomiting	CSDH	Craniotomy	Microsurgery	Clinical recovery
Patel [102]	1	9/M	B/I	None	Headache, vomiting	CSDH	Craniotomy	Microsurgery	Clinical recovery
Pillai [104]	2	23/M	U (left)/III	Head trauma	Headache, nausea	CSDH	Burr holes	None	Clinical recovery
		41/M	U (right)/II	Head trauma	Headache, nausea	CSDH	Burr holes	None	Clinical recovery
Poirrier [105]	1	15/M	B/II	None	Headache, vomiting, blurred vision	Hygroma	Burr holes; subdural-peritoneal shunt	None	Hygroma resolution; increase in cyst size
Prabhu [106]	1	16/F	U (left)/II	Head trauma	LOC	CSDH	Craniotomy	Microsurgery	Hematoma resolution, cyst reduction
Prokopienko [107]	1	36/M	-	Head trauma	-	IH	-	-	-
Rajesh [108]	1	15/M	U (left)/II	Minor head trauma	Headache, vomiting, papilledema with blurred vision	Bilateral subdural hygromas	NR	NR	NR
Rakier [109]	1	13/F	U (left)/II	None	Acute headache	None	None	None	Cyst resolution after spontaneous rupture
Rogers [110]	6	10/F	U (left)	Head trauma	Papilledema	CSDH	Burr holes, craniotomy	Microsurgery, cystoperitoneal shunt	Clinical recovery
		11/M	U (right)/III	Head trauma	Papilledema, VI CN palsy	CSDH	Burr holes	None	Clinical recovery
		12/F	U (left)	Head trauma	Papilledema	CSDH	Burr holes, craniotomy	Microsurgery, cystoperitoneal shunt	Clinical recovery
		6/M	U (right)	None	Papilledema, proptosis	CSDH	Craniotomy	Microsurgery, cystoperitoneal shunt	Clinical recovery
		6/F	U (right)	None	Papilledema	CSDH	Craniotomy	Microsurgery	Clinical recovery
		6/M	U (left)	Head trauma	Papilledema	CSDH	Burr holes, craniotomy	Microsurgery, cystoperitoneal shunt	Clinical recovery
Seddighi [111]	1	23/M	U (left)/III	Head trauma	Headache, vomiting, papilledema	Epidural hematoma	Craniotomy	Microsurgery	Clinical recovery
Sener [112]	4	10–18/80% M, 20% F	U (left)	60% head trauma	Headache (100%)	ASDH (80%), subdural hygroma (20%)	NR	NR	NR
Servadei [113]	3	42/M	U (left)	Head trauma	Headache	ASDH	Craniotomy	Microsurgery	Hematoma resolution, cyst persistence
		19/M	U (left)	Head trauma	Headache, vomiting	ASDH	Craniotomy	None	Hematoma resolution, cyst persistence
		64/M	U (right)	Head trauma	Headache	CSDH	Craniotomy	None	NR
Shresta [114]	4	21/M	U (left)/III	None	Headache	CSDH	Craniotomy	Microsurgery	Cyst and hematoma resolution
		15/M	U (left)	None	Headache	CSDH	Craniotomy	Microsurgery	Clinical recovery
		16/F	U (left)	None	Headache, dizziness	CSDH	Craniotomy	Microsurgery	Clinical recovery
		5/F	U (right)	None	Headache, vomiting	CSDH	Craniotomy	Microsurgery	Cyst and hematoma resolution
Singh [16]	1	11/F	U (right)/II	None	Headache, papilledema, photophobia	Subdural hygroma	Craniotomy	Microsurgery	Clinical recovery
Slaviero [115]	2	15/M	U (left)/II	Head trauma	Headache, vomiting, confusion	CSDH	Burr hole	Endoscopic fenestration	Hematoma resolution, cyst persistence
		5/M	U (left)/II	None	Headache, drowsiness	Hygroma	Burr hole	Endoscopic fenestration	Hematoma resolution, cyst reduction
Sprung [20]	60	1–82/76% M, 24% F	58% U (left); 33% U (right); 9% B	71%	90% headache; 15% drowsiness; 37% focal neurologic signs	Subdural effusion (76% ipsilateral, 5% contralateral, 10% bilateral)	47% burr hole; 22% craniotomy; 12% endoscopy; 5% subduro-peritoneal shunt; 10% conservative treatment; 4% not reported		Hematoma: 92% complete regression; 8% incomplete regression; Cyst: 40% no change, 40% incomplete regresion, 4% complete regression, 16% increase
Tasar [118]	2	-	-	-	-	CSDH	-	-	-
Takayasu [116]	2	8/M	U (left)/III	Head trauma	Headache, nausea, asthenia	CSDH	Craniotomy	Microsurgery	Hematoma resolution, cyst reduction
		3/M	U (left)/III	Head trauma	Headache	CSDH	Burr hole	None	Hematoma resolution, cyst reduction
Takizawa [117]	12	8–71/92% M, 8% F	75% U (left), 25% U (right)	83% head trauma	100% headache; 8% focal neurologic signs; 8% coma	75% ASDH, 8% CSDH, 17% NR	25% craniotomy, 75% burr holes	25% microsurgery, 75% none	75% clinical recovery; 25% NR
Tinois [119]	10	5–16/M	5—I 5—II	Head trauma	-	SDH	SPS	2 microsurgery 1 CPS 7 none	Complete resolution
Tsitsopoulos [120]	1	15/M	U (left)/III	Head trauma	Headache	CSDH	Craniotomy	Microsurgery	Hematoma resolution, cyst persistence
Ulmer [121]	1	43/M	U (left)/III	Mild sport trauma	Headache, nausea	CSDH	Craniotomy	Microsurgery	Clinical recovery
Van Der Meche [90]	4	16/M	U (NR)	Minor head trauma	Headache, nausea, vomiting	CSDH	Craniotomy	Microsurgery	Clinical recovery
		7/M	U (NR)	Minor head trauma	Headache, diplopia, papilledema	CSDH	Craniotomy	Microsurgery	Clinical recovery
		10/F	U (NR)	Minor head trauma	Headache, nausea, vomiting	CSDH	Craniotomy	Microsurgery	Clinical recovery
		15/F	U (NR)	Minor head trauma	Headache, nausea, vomiting, papilledema	CSDH	Craniotomy	Microsurgery	Clinical recovery
Varma [122]	6	24/M	U (right)	Sport accident	Headache, vomiting	Hygroma	Craniotomy	Microsurgery	Clinical recovery
		7/M	U (right)	Head trauma	Headache, vomiting	Hygroma	Craniotomy	Microsurgery	Clinical recovery
		7/M	U (left)	Head trauma	Headache, vomiting, hemiparesis, papilledema	Hygroma	Craniotomy	Microsurgery	Clinical recovery
		17/M	U (right)	Sport accident	Headache, vomiting, hemiparesis, papilledema	Hygroma	Craniotomy	Microsurgery	Clinical recovery
		21/M	U (right)	Head trauma	Headache, vomiting, hemiparesis, papilledema	CSDH	Craniotomy	Microsurgery	Clinical recovery
		13/M	U (right)	Sport accident	Headache, vomiting, papilledema	CSDH	Craniotomy	Microsurgery	Clinical recovery
Wu [123]	14	1–41/93% M, 7% F	57% U (left), 36% U (right), 7% B	50% head trauma	NR	CSDH (7% bilateral)	71% burr holes, 29% craniotomy	29% microsurgery	100% clinical recovery
Yamauchi [124]	1	7/M	U (right)/II	None	Increased head circumference	None	None	None	Spontaneous disappearance of the cyst
Yoshioka [125]	1	0.1/M	U (left)/III	None	None	None	None	None	Spontaneous disappearance of the cyst after meningitis
Yuksel [126]	1	17/M	U (left)/II	None	Headache, VI CN palsy, papilledema	CSDH	Craniotomy	None	Hematoma resolution, cyst persistence
Zeng [127]	2	14/M	U (left)/III	Sport accident	Headache, vomiting, hemiparesis	CSDH	Craniotomy	Microsurgery	Clinical recovery
		17/M	U (left)/II	Sport accident	Headache	CSDH	Craniotomy	Microsurgery	Hematoma resolution, cyst persistence
Zhang [128]	2	21/M	U (left)/II	None	Headache, vomiting	CSDH	Craniotomy	Microsurgery	Clinical recovery
		9/F	U (left)/II	None	Headache, vomiting	CSDH	Craniotomy	Microsurgery	Clinical recovery
Ziaka [129]	1	38/M	B/II	None	Headache	CSDH	Craniotomy	Microsurgery	Clinical recovery
Ziyal [130]	1	26/M	-	-	-	CSDH	-	-	-

Abbreviations: *EDH*, epidural hematoma; *CSDH*, chronic subdural hematoma; *ASDH*, acute subdural hematoma; *SDH*, subdural hygroma; *IH*, intracystic hemorrhage; *B*, bilateral; *U*, unilateral; *LOC*, loss of consciousness

**Table 2:** Review of the literature analysis

**Etiology**	**Number of patients**
Trauma	226
Spontaneous	106
Not specified	98
**Age**	**Number of patients**
< 18	203
> 18	69
Not specified	159
**Sex**	**Number of patients**
Male	215
Female	54
Not specified	161
**Galassi classification**	**Number of patients**
I	42
II	105
III	69
Not specified	213
**Complication of rupture**	**Number of patients**
Subdural collection (hygroma, CSDH, ASDH)	347
Epidural collection	11
Intracystic hemorrhage	28
Not specified	44
**Complication treatment**	**Number of patients**
Burr holes	115
Craniotomy	113
Shunt	41
Medical treatment	36
Not specified	86
**Cyst treatment**	**Number of patients**
Endoscopic fenestration	20
Microscopic fenestration	113
Shunt	19
No treatment	96
Not specified	133

*CSDH*, chronic subdural hematoma; *ASDH*, acute subdural hematoma

### Personal series

The series is composed of 16 children (M/F ratio: 4.3) with an age at diagnosis ranging from 9 months to 16 years (average: 8.7 years). They account for 15.8% of all SACs (101 cases) and 8.7% of intracranial arachnoid cyst (183 cases) admitted to our institution in the same time period. They also represent about one-fourth of the fluid subdural collections operated on in this period.

A history of (head) injury was reported in 75% of cases. The left side (68.7%) and the Galassi type II (50%) were predominant among SACs. Signs/symptoms of raised ICP were largely prevalent; only one patient was asymptomatic at the admission (6%).

Subdural hygroma occurred in 12 children (75%) and was managed by evacuation through burr hole and external drainage in all cases, plus subduro-peritoneal shunt (SP shunt) in 7 cases for persisting hygroma. Subdural hematoma occurred in the remaining 4 cases (25%) and was managed by burr hole evacuation in all but one patient who required a mini-craniotomy because of intracystic hemorrhage. All patients recovered from their symptoms after the subdural collection evacuation. All patients received clinical and MRI follow-up other than (brain SPECT or) perfusion MRI; only one cases needed ICP recording because of ghost-cyst syndrome.

After a mean 9.8-year follow-up (range: 2.5–16.2 years), all patients are asymptomatic (apart form one who developed migraine). Two children had a permanent cyst regression after the evacuation of the subdural collection. Out of the remaining 14 patients, 7 children (43.7%) underwent endoscopic (4 cases) or microsurgical cyst fenestration (3 cases), with cyst reduction in all cases. No surgical complications occurred except for transient CSF leakage in case # 11. Three out of 7 patients needed a permanent SP shunt. The cyst of not operated on patients remains unchanged or reduced in size. The results are summarized in Table 3.

**Table 3:** Personal series

**No**	**Sex, age**	**Trauma**	**Side, Galassi’s type**	**Symptoms**	**Subdural fluid collection and management**	**Cyst management**	**Clinical outcome**	**Radiological outcome**	**FU (years)**
1	M, 16 years	Not reported	Left, grade III	Seizures	Hygroma: burr hole + SP shunt	Endoscopic fenestration + shunt removal	Asymptomatic, seizure regressed	Cyst reduced, hygroma disappeared	12.1
2	M, 13 years	Yes	Left, grade II	Sudden headache	Hematoma: burr holes hygroma SP shunt	None (cyst effaced) + shunt removal	Asymptomatic	Cyst and hygroma disappeared	16.2
3	M, 11 years	Yes	Right, grade III	Acute left hemiparesis, left side paresthesia, headache, vomiting	Hygroma: burr hole + SP shunt	Endoscopic fenestration + SP shunt maintenance (persistent hygroma)	Asymptomatic, hemiparesis regressed	Cyst and hygroma reduced (both persistent)	13.2
4	M, 10 years	Yes	Left, grade II	Headache, vomiting	Hematoma: burr holes	None	Asymptomatic	Cyst unchanged, hematoma disappeared	15
5	M, 4 years	Yes	Left, grade III	Incidental finding at CT scan after head injury (no symptoms)	Hygroma: burr hole + SP shunt	None + shunt removal	Asymptomatic	Cyst unchanged, hygroma disappeared	12.8
6	M, 1 year	Yes	Left, grade II	Transient post-trauma loss of consciousness with quick recovering; macrocrania	Hygroma: burr hole	Microsurgical cyst fenestration	Asymptomatic	Cyst almost effaced, hygroma disappeared	12.4
7	F, 14 years	Not reported	Right, grade II	Headache, vomiting, diplopia	Hematoma: burr holes	None	Asymptomatic	Cyst unchanged, hematoma disappeared	15
8	M, 6 years	Yes	Right, grade III	Headache, lethargy	Hygroma: burr holes	None	Asymptomatic	Cyst slightly reduced, hygroma disappeared	11.1
9	M 9 mts	Not reported	Left, grade III	Irritability, bulging fontanel; macrocrania	Hygroma: burr holes + SP shunt	None + SP shunt maintenance (persistent hygroma)	Asymptomatic (sporadic headache)	Cyst reduced, hygroma almost disappeared but shunt dependency	11.8
10	M, 8 years	Yes	Left, grade II	Headache, lethargy	Hygroma: burr holes	None	Asymptomatic	Cyst unchanged, hygroma disappeared	9
11	M, 5 years	Not reported	Left, grade III	Headache, diplopia	Hygroma: burr holes	Microsurgical fenestration	Migraine	Cyst reduced, hygroma disappeared	7.9
12	M, 16 years	Yes	Right, grade II	Headache, vomiting	Subacute subdural hematoma and intracystic hemorrhage: mini-craniotomy	Microsurgical fenestration	Asymptomatic	Cyst reduced, hematoma disappeared	8.5
13	F, 13 mts	Yes	Left, grade II	Lethargy, vomiting	Hygroma: burr hole	None	Asymptomatic	Cyst unchanged, hygroma disappeared	6.3
14	M, 15 years	Yes	Right, grade II	Headache	Hygroma: conservative treatment	Endoscopic fenestration	Asymptomatic	Cyst reduced, hygroma disappeared	3.5
15	F, 13 years	Yes	Left, grade II	Headache, lethargy	Hygroma: burr hole + SP shunt	Endoscopic fenestration + shunt removal	Asymptomatic	Cyst reduced, hygroma disappeared	2.5
16	M, 6 years	Yes	Left, grade III	Headache, vomiting	Hygroma: burr hole + SP shunt	None ICP monitoring	Asymptomatic (sporadic headache)	Cyst effaced, hygroma disappeared, ghost cyst syndrome: VP shunt	3

### Exemplary case

**Fig. 1:**
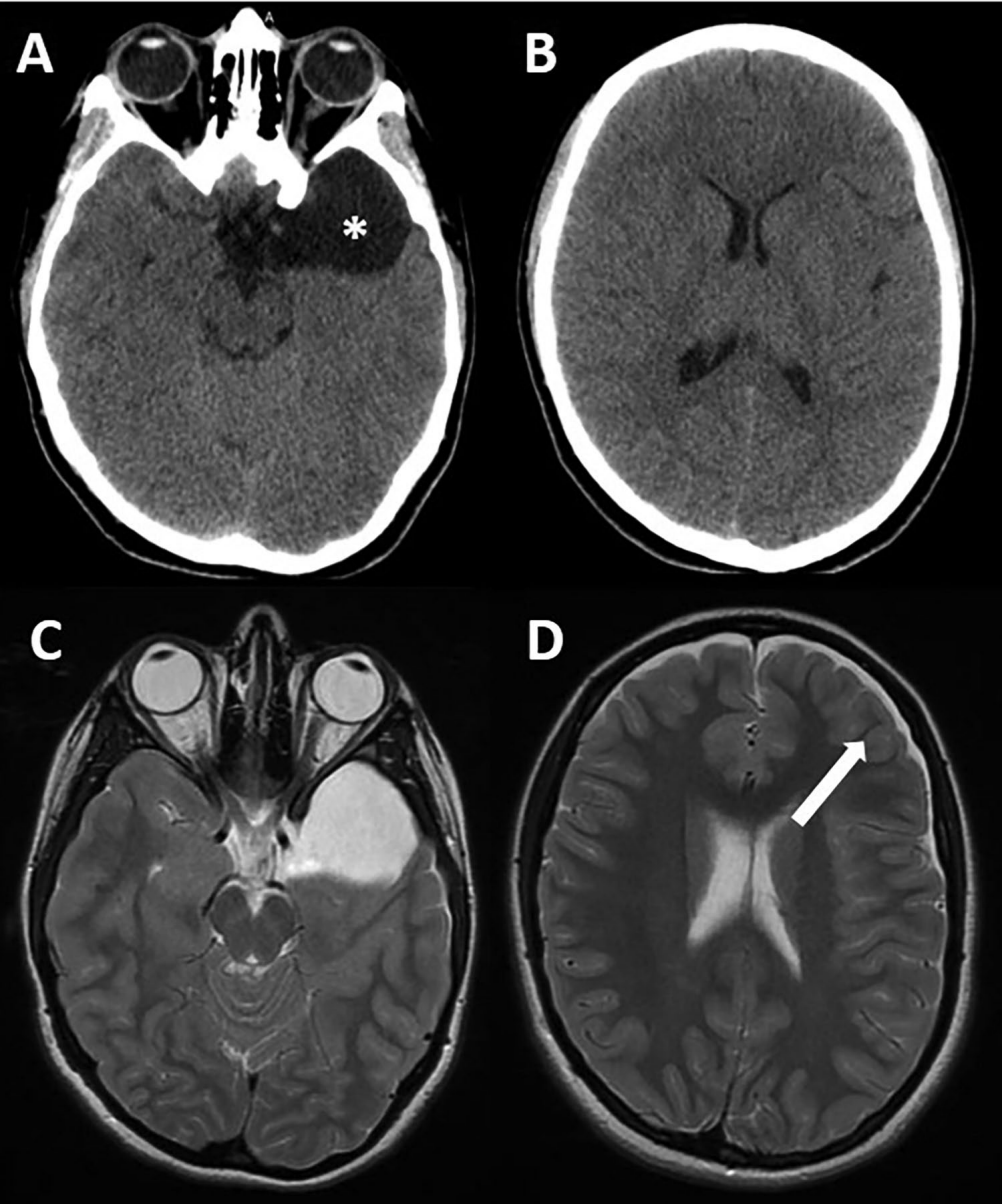
**A**, **B** CT scan showing the left SAC (asterisk); **C**, **D** MRI (axial T2 view) showing the persistence of the cyst and the appearance of a hygroma (arrow)

**Fig. 2:**
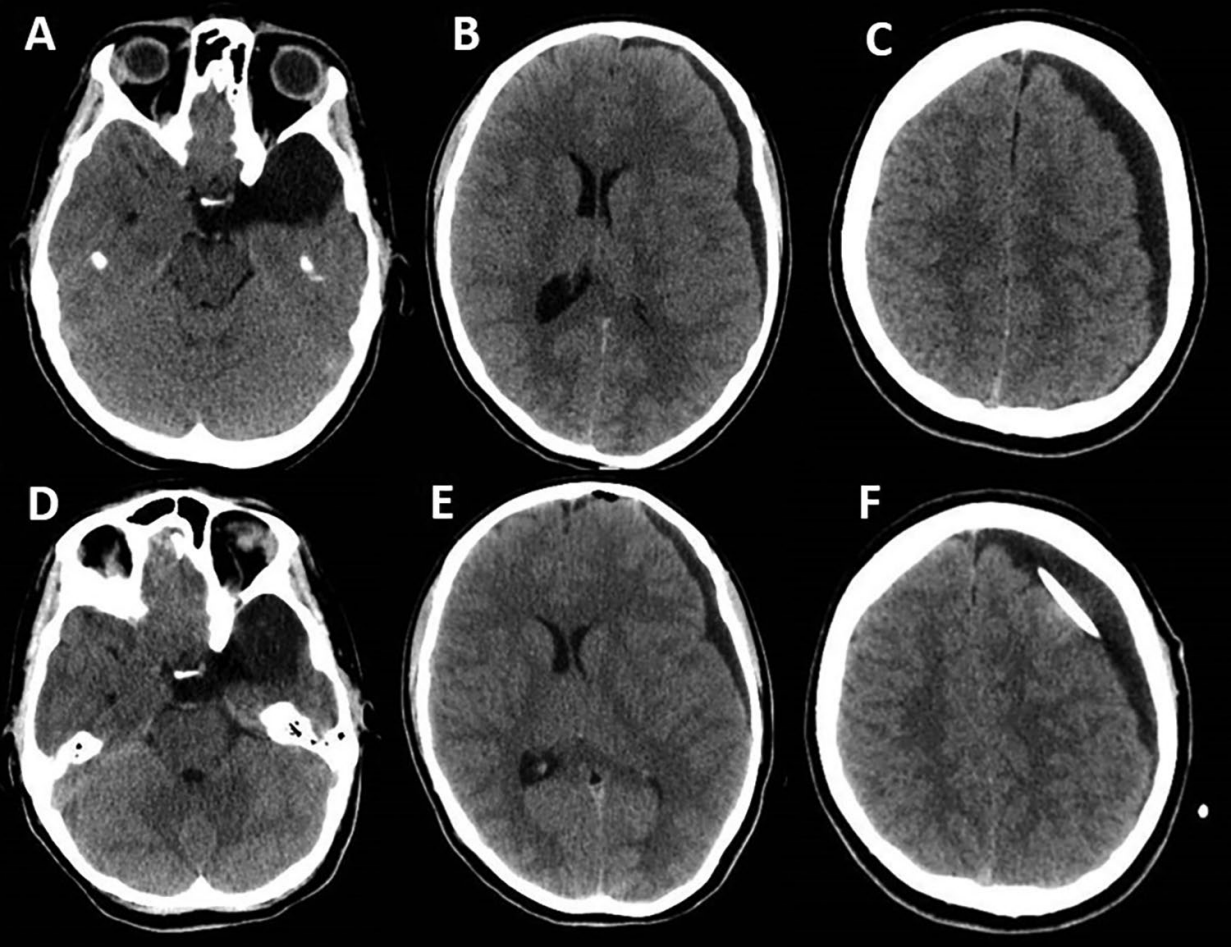
**A**–**C** CT scan showing the increase of the hygroma; **D**–**F** CT scan performed a few days after the placement of a subduro-peritoneal shunt, with no significant changes compared with the previous one

**Fig. 3:**
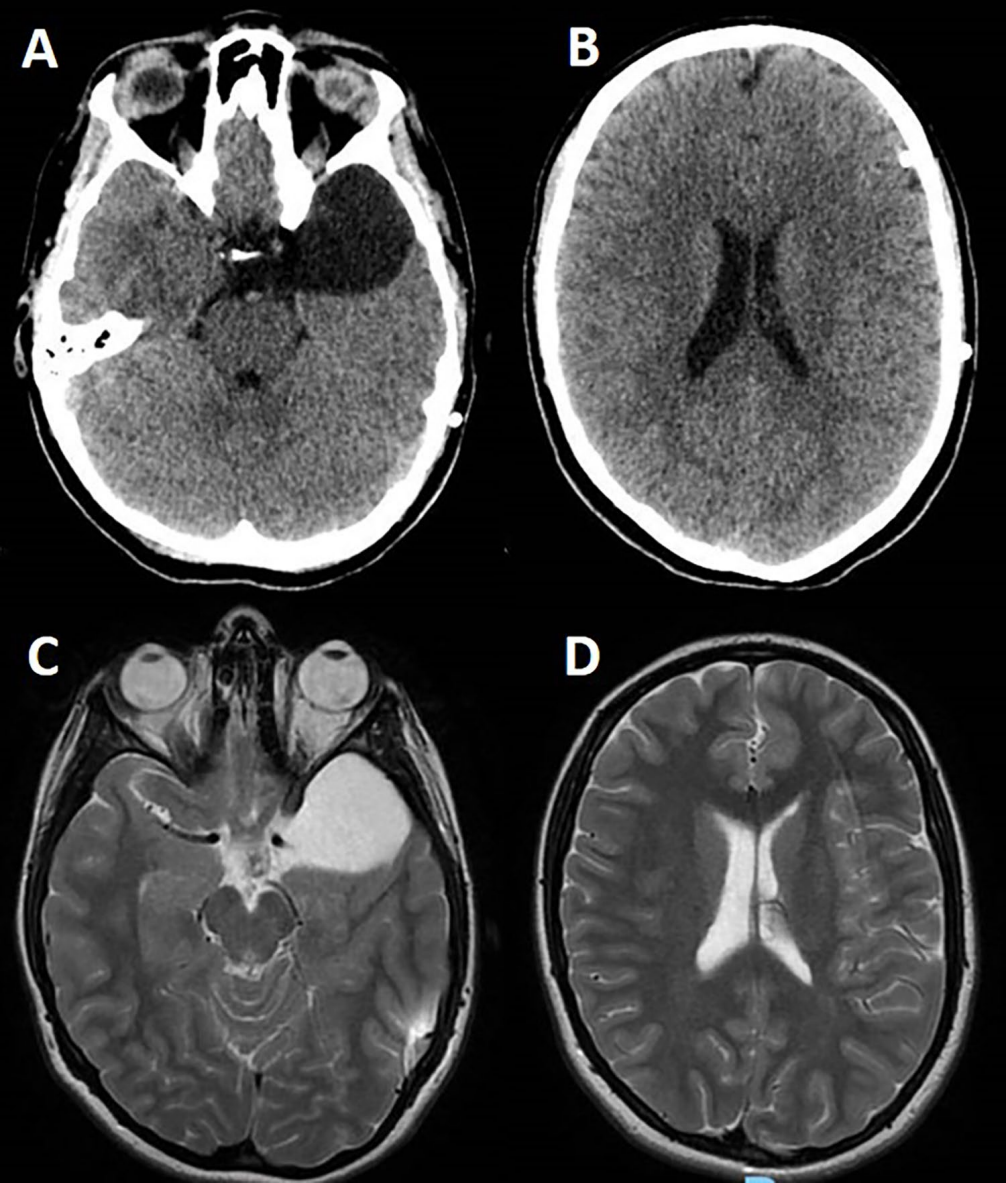
CT scan (**A**, **B**) and MRI (**C**, **D**) showing the complete resolution of the hygroma. The cyst is unchanged

**Fig. 4:**
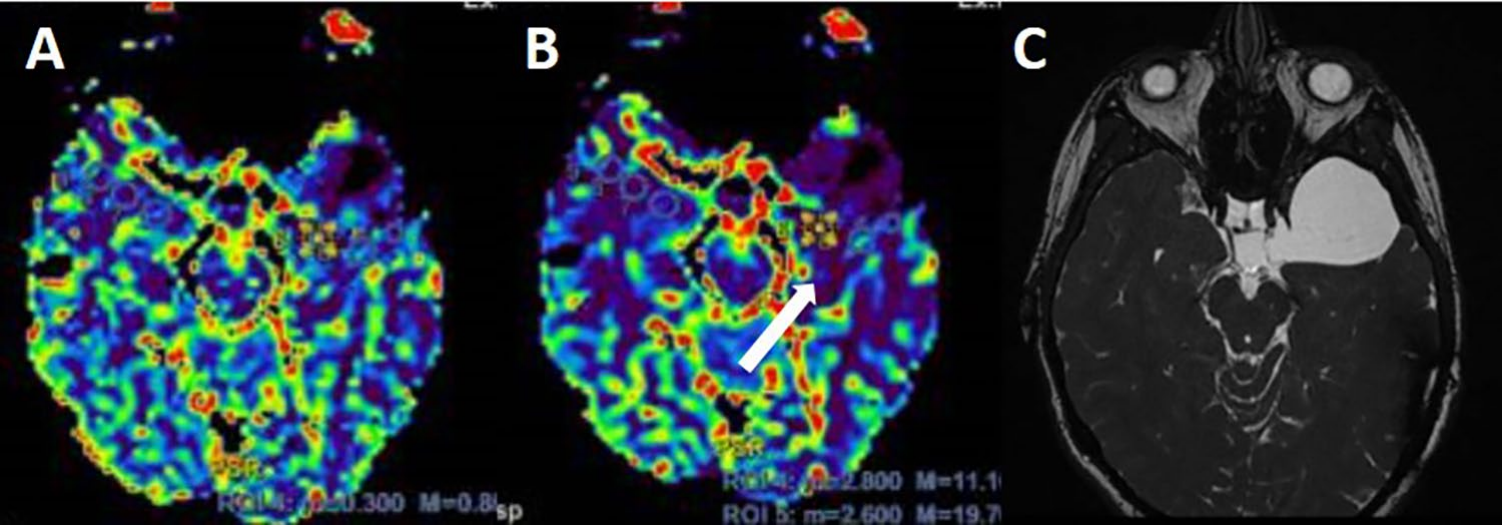
Perfusion MRI (**A**, **B**) showing a decrease of the signal in the temporal brain surrounding the SAC. The MRI with FIESTA sequences points out no communication of the cyst with the basal cisterns and, at the same time, enough room for a surgical fenestration (**C**)

**Fig. 5:**
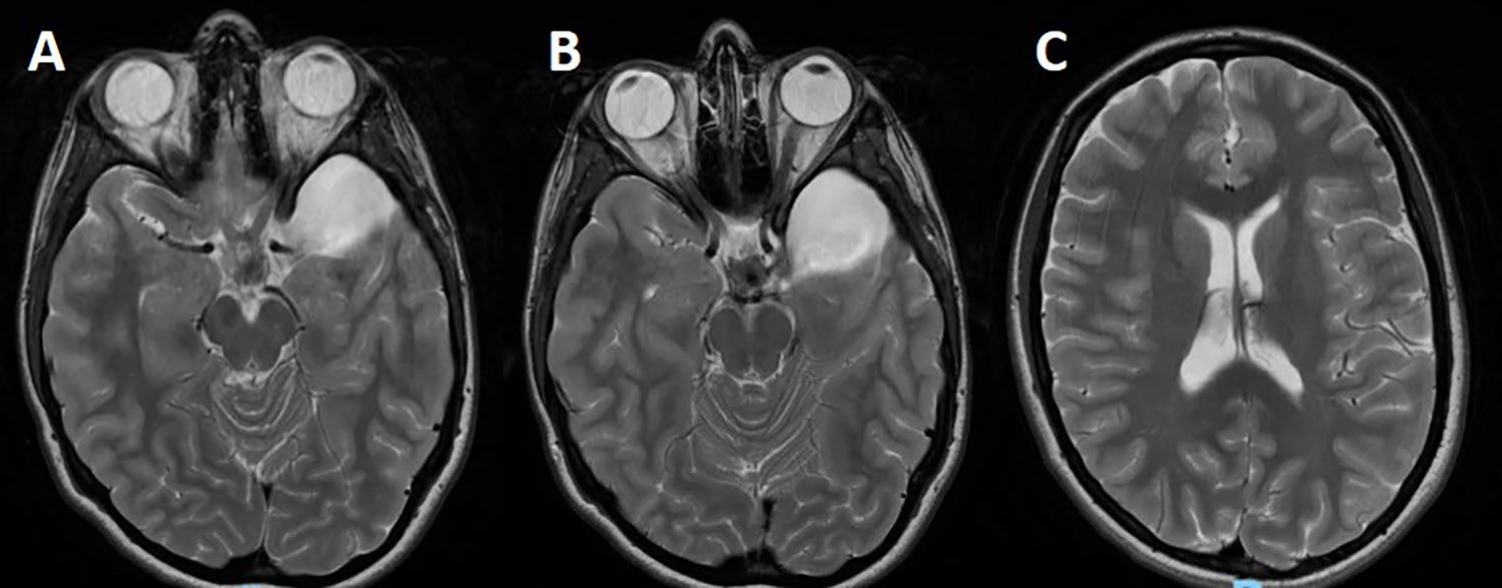
MRI, realized about 2 years from the onset of the clinical history, showing a slight decrease of the cyst volume and the flow artifact indicating the fenestration (**A**, **B**). No recurrence of the hygroma is evident (**C**)

This previously asymptomatic 13-year-old girl was admitted to our institution following a head injury (car accident). A short concussion was reported but no neurological deficit was evident at the physical examination performed at the admission. A CT scan did not show acute sequelae of the trauma but revealed a Galassi type I left SAC (Fig. 1A, B). The day after the injury, the patient developed headache. An MRI was then performed, which showed the appearance of a thin hygroma, suggesting a rupture of the cyst (Fig. 1C, D). Twenty-four hours later, the headache became intense and vomiting and lethargy appeared. A new CT scan demonstrated a significant increase of the hygroma (Fig. 2A–C). The patient underwent a burr hole evacuation of the hygroma in emergency (an external drainage was left in place) with quick and complete clinical recovery. Afterward, a subduro-peritoneal shunt (adjustable valve) was placed because of the persistence of the hygroma (Fig. 2D–F). In the next months, the valve setting pressure was progressively elevated up to the maximum level (20 cm H2O). The patient was followed-up clinically and radiologically. She remained asymptomatic. The neuroimaging studies performed 1 year after the shunt implantation (and 3 months after the shunt “closure”) demonstrated the resolution of the hygroma, while the cyst was unchanged (Fig. 3). A perfusion MRI showed an asymmetric signal of the temporal brain with hypoperfusion of the brain surrounding the cyst compared with the contralateral region (Fig. 4A, B). The possibility of a good endoscopic fenestration was assessed by FIESTA MRI (Fig. 4C). Finally, the patient underwent an endoscopic fenestration of the SAC into the basal cisterns, with a good clinical and radiological outcome (Fig. 5).

## Discussion

### Epidemiological considerations

A first goal of this review was to estimate the “epidemiological” impact of the rupture of SACs and the weight that this event can have in the clinical practice. Of course, a real epidemiological study on this topic is not possible because of the rare and often incidental diagnosis of SACs. Therefore, population-based studies are hard to obtain due to the lack of systematic screenings on the population and the absence of a relevant clinical picture that can orientate these investigations. Moreover, most of the available studies include mixed series, which may prevent a reliable epidemiological evaluation (they often consist of both SACs (higher risk of rupture) and cysts located elsewhere (lower risk), and/or both children (higher risk) and adults (lower risk), and/or both symptomatic and asymptomatic patients), or isolated cases, where the prevalence of the rupture cannot be specified due to the lack of a population denominator. However, some conclusions on the clinical impact can be deduced based on the relatively large number of collected cases, which also reveal that SACs seem to be not rare as commonly thought.

As expected, the series considering symptomatic subjects point out an extremely variable incidence of rupture, ranging from 2 to 55% [5, 94, 100]. The main bias is that they tend to overestimate the phenomenon because most of the symptomatic patients experience symptoms just because of the rupture of the cyst. More reliable information may be taken from hospital-based studies analyzing large portions of the population. Parsch et al. detected 94 SACs among 11,487 brain MRIs of asymptomatic patients of all ages [100]. Only two cases showed the signs of rupture. The risk of rupture was calculated to be as high as 0.04% per year in asymptomatic patients, which is lower than that usually considered (≤ 0.1% per year) [67, 106, 131]. The authors also reviewed the reports of 658 symptomatic patients with subdural hematoma/hygroma and observed a 2.4% incidence of arachnoid cysts (16 cases) among them, thus confirming the higher rate of rupture among symptomatic patients. Similar figures have been reported by other authors [18, 46, 123]. Mori et al., for example, found a ruptured arachnoid cyst in 12 out of 541 patients (both children and adults) admitted for a subdural fluid collection (2.2%); 8 out of 12 patients showed SACs (6 were children) [7].

The first attempt to estimate the relationship between SACs and their rupture was made in 1988 by Galassi et al. who observed a chronic subdural hematoma in 7 of their 77 patients with SACs (9%) [52]. Wester and Helland afterward reported a 4.6% incidence among all intracranial cysts (11 out of 241 cases) and a 6.5% incidence among SACs (11 out of 174 cases) [132]. The results of the present series match these data, with a 15.8% incidence of rupture among SACs (8.7% among all intracranial arachnoid cysts) and with ruptured SACs representing 25% of all operated on subdural fluid collections. The higher rate of incidence of rupture among SACs and ruptured SACs among subdural collections compared with the literature depends on the purely pediatric population. Children, indeed, are more prone to develop a rupture than adults and to present SACs [7, 18, 100]. Moreover, subdural collections in children are usually a complication of a disease (arachnoid cyst, AVM, shaken baby syndrome, etc.) rather than from a mild head injury as it happens in the elderly. In the series provided by Mori et al., ruptured SACs were associated with subdural collection in 65% of children and in only 1.3% of adults [7].

These figures confirm some epidemiological aspects clearly emerging from the literature (Table 1). First of all, although it is frequently reported as “spontaneous,” the rupture is more often a post-traumatic event. About a half of the 430 collected cases (52.5%) are actually associated with a clear trauma. A (relatively) mild injury, however, could be misdiagnosed in part of the remaining cases where this event was not found (28.5%) or not reported (22.5%) (Table 2). Secondly, SACs undergo rupture more frequently than cysts located elsewhere [30, 132–134]. According to the comparative series available, the risk of rupture of SACs is twofold or even four times higher than other arachnoid cysts [7, 23, 94, 112]. Thirdly, the rupture occurs more commonly in boys than in girls (50% male, 13% female, 37% not specified) (Table 1). Such a significant difference mainly results from the higher incidence of SACs in the male sex [4]. Moreover, it could reflect also the higher predisposition to injury usually showed by boys compared with girls. This would also explain another significant difference emerging from the literature, which is the higher incidence of rupture among the young population, namely children. Indeed, even though the patients’ age was not reported in about a half of the papers, patients younger than 18 years were involved twice more than those older than 18 years (203 vs 67 cases) (Table 2). It is worth noting that many among the > 18-year-old patients were young adults (Table 1).

In summary, although the real incidence of the phenomenon cannot be calculated, this review suggests that SACs rupture is a rare event in the general population (0.04%) but it is not negligible when considering the population affected by SACs (around 6–10%), especially in children (up to 15%). Young age, male sex, and history of (head) trauma are the main epidemiological risk factors. It is worth noting that these figures concern series focusing on ruptured SACs. If all arachnoid cysts in all patients with head injury are considered, the phenomenon of rupture is very rare. In their analysis on 68 children affected by arachnoid cysts and suffering from blunt head injury (0.4% out of 15,899 patients receiving head CT scan for traumatic injury), no rupture of arachnoid cyst was observed [135].

### Etio-pathological hypotheses

The rupture of SACs would result from the impact of the cyst against the ipsilateral sphenoidal wing or by the traumatic force directly acting on the cyst because of a thinned temporal bone [18, 23]. The cause is usually represented by a head injury, often occurring during sport activities [10, 27, 30, 48, 60, 106, 136]. As mentioned, a traumatic event is reported “only” in a half of the patients but the relatively high rate of “spontaneous” ruptures might depend on misdiagnosed, mild traumas, or on events that are not considered traumatic (e.g. cough, Valsalva maneuver, physical exercise) [16, 30, 39]. Actually, even an abrupt movement of the head could cause the impact of the cyst against the sphenoid wing, thus causing its rupture. According to some authors, who observed that subdural collections are rare after temporal lobectomy, this phenomenon would depend on the reduced compliance of SACs against the impacts compared with the brain [110]. Therefore, although it cannot be excluded (e.g., peak of blood hypertension), a really spontaneous rupture should be considered an exceptional event.

Several hypotheses have been formulated to explain the formation of a subdural fluid collection after the cyst rupture. As for the chronic hematoma of the elderly, many authors consider the rupture of small leptomeningeal vessels of the cyst wall or of small bridging veins crossing the cyst the main cause of the subdural and/or intracystic bleeding [13, 23, 67, 100, 134]. The break of the bridging veins would justify also the occurrence of a contralateral subdural hematoma reported by some authors [104, 119]. The rupture of this veins is favored by the lack of the solid support provided by the brain tissue that makes easy the vein tearing even after minor injuries in young patients [67]. Wester and coworkers verified the presence of bridging veins crossing the external and internal surfaces of the cyst and hypothesized that their rupture depends on the weak adhesion of the cyst wall to the overlying dura mater with subsequent easy detachment of the wall in case of head injury and bleeding from the inner dural surface [5]. Other authors hypothesized that the cyst rupture first results in the formation of a hygroma that produces tearing of the bridging veins and small bleedings, which, in turn, cause the chronic subdural hematoma [7, 105]. This theory would explain why subdural hematomas are more frequent in older patients (young adults and, namely, adults) while hygromas are more common in children (Table 1). Children, indeed, are prone to become acutely symptomatic due to their trophic brain, thus preventing the chronic hematoma formation. It is a common experience, in the clinical practice, to find a variable amount of blood diluted in the hygroma of a ruptured pediatric SAC. Actually, hygroma is thought to originate from the laceration of the cyst wall after contact with the sphenoidal wing as well, with subsequent CSF escape into the subdural space [7, 16, 23]. Such a communication between the intracystic and the subdural space was detected during surgery by some authors in the past [39, 40, 100]. Two further hypotheses have been formulated to explain the occurrence of the hygroma: 1) The communication between the cyst and the subarachnoid space would follow a minor head injury causing a flap-valve mechanism with a CSF flow from the subarachnoid space into the cyst. This would result in an increase in size and pressure of the cyst with rupture into the subdural space; 2) the Valsalva maneuver would cause a transient raise of ICP with rupture of the cyst into the subarachnoid space [55]. When the cyst wall is richly vascularized, the rupture may result in a “mixed” hygroma or an acute subdural hemorrhage and/or an intracystic hematoma. Differently from the hematoma, the presence of a hygroma contralateral to a SAC should suggest the presence of a (small) contralateral ruptured SAC, too [95].

The occurrence of an extradural hematoma is probably the result of the dural detachment due to the sudden decompression following the cyst rupture [100, 121]. Some authors propounded the stripping of the dura from the inner skull table as the cause of the tear in the middle meningeal vein to explain the low-pressure extravasation usually occurring in such instances [29]. Abbas et al., finally, hypothesized that the thinned temporal bone surrounding the cyst, being more fragile and prone to be fractured, could favor the bleeding from the meningeal vessels and the detachment of the dura in case of moderate head injury, thus leading to an extradural hematoma [21].

As far as possible etiologic risk factors are concerned, no correlation between cyst volume and risk of rupture has been clearly demonstrated by the personal series (Table 3) and by several authors in the past [106, 131, 137]. However, the present extensive review of the literature shows that Galassi type II (24.5%) and type III (16%) cysts are more likely to undergo rupture than Galassi type I (10%), based on the articles reporting this information (50.5% of the analyzed papers) (Tables 1 and 2). This would suggest the larger the volume, the higher the risk of rupture or, as an alternative option, that the absence of bony scalloping as well as the lesser number “floating” vascular structures in Galassi type I cysts could prevent the effects of the traumatic forces. According to the case–control study on 29 cases by Cress et al., the cyst volume (namely, more than 5 cm of diameter) correlated with the risk of cyst rupture as well as the occurrence of a recent head injury [6]. Moreover, other authors pointed the local thinning of the bone (scalloping) as risk factor for the cyst rupture because a bone fracture could cause a direct break of the cyst wall [18]. According to others, the so-called subdural compartment, a potential space resulting from the invasion of the arachnoid cyst wall by dural borders cells, has been postulated to be a “weakness point” favoring the cyst rupture even after mild injuries [46]. A similar mechanism has been advocated for possible micro-adhesions between SAC and subdural space [100]. Finally, some vascular anomalies (e.g., abnormal and fragile vessels surrounding the cyst, missing Sylvian superficial vein, and spheno-parietal sinus) have been hypothesized to concur to the bleeding after the rupture [106, 110, 138].

### Clinical aspects

The presentation of ruptured SACs is mainly characterized by worsening raised ICP symptoms in a previously asymptomatic patient, often with apparently normal neuropsychological development [60, 139]. Typically, raised ICP occurs some hours/days or even weeks after the injury (time necessary for the subdural collection formation) [11, 13]. Headache and vomiting are almost invariably reported, sometimes in association with papilledema and/or visual deficits (Table 1). In small children, as expected, also macrocrania and bulging fontanels can be found [32]. On the other hand, focal deficits (as hemiparesis or cranial nerve palsy) are less common [9], complicating the clinical picture in 10% and 6% of cases in the overall literature and in the present series, respectively (Tables 1 and 3). However, when considering large series, focal neurological deficits are found to range from 8 to 37% [20, 100, 117]. Seizures are occasionally reported, affecting at most 6% of cases [100]. A transient loss of consciousness immediately after the head injury is not rare [21, 23, 29, 37, 52]. In 27 cases, the brain concussion and the rupture of the SACs were complicated by coma [88, 117].

According to the data available from the literature and the present series, the mean age at onset is 19 years, ranging from newborns to 76-year-old patients. Such a relatively young age accounts for the exceptional occurrence of asymptomatic ruptured SCAs (although it cannot be excluded that several asymptomatic cases are not diagnosed and, thus, not reported). One asymptomatic case was detected in the personal series and only 3 (0.7%) in the literature [7, 8, 15]. One could speculate that the absence of symptoms in these few cases depends on (post-traumatic or pre-existent) micro-fenestrations of the arachnoid cyst into the basal cisterns, which ensures CSF escape counterbalancing the mass effect of the subdural collection.

In the great majority of cases, the SCA rupture leads to a subdural collection (Table 2). This kind of complication was reported in 80% of the collected cases from the literature (347/430 patients), but it was as high as 91% if considering only the patients with complete information on the rupture’s consequences (391/430 patients) (Table 2). More in detail, out of 446 cases (literature plus personal series), 142 patients presented a chronic subdural hematoma (33%), 157 a subdural hygroma (36.5%), 28 a chronic subdural hematoma plus intracystic bleeding (6.5%), 20 an acute subdural hematoma (4.5%), 11 an extradural hematoma (2.5%), 28 a purely intracystic bleeding (6.5%), and two an acute subdural hematoma plus intracystic bleeding (0.5%). In the remaining 5 cases, the SAC disappeared after the head injury (1.5%). These figures suggest the following conclusions: 1) The subdural fluid collection is the typical complication of the SAC rupture, independently from SAC’s side and size, type of injury, and patient’s age, the hygroma being slightly predominant compared with the hematoma; 2) the distinction between chronic hematoma and hygroma does not affect the clinical impact, since the management is similar (burr holes evacuation), except for sporadic cases of subdural hematoma treated by craniotomy. A difference in the management can occur in case of bilateral or contralateral subdural effusions although, among the subdural collections (391 cases), only 13 of them were bilateral (3.3%) and 3 contralateral to the SAC (0.7%); 3) the rate of complications with a possible major clinical impact, namely acute subdural hematoma, extradural hematoma, and intracystic bleeding, is not negligible (89 out of 430 cases, 21%). However, even though these complications usually need a craniotomy for their resolution, they can be managed even by a conservative treatment in favorable cases; 4) only one-third of intracystic bleeding occurs as isolated, while in two-third of cases, this bleeding is coupled with subdural effusions. The rhexis of the small vessels surrounding the cyst could account for the purely intracystic bleeding after the rupture, while the simultaneous cyst rupture and bridging vein tears would justify the association between subdural collection and intracystic hemorrhage; 5) SAC disappearance after head injury is a rare but not exceptional phenomenon. The advocated mechanisms for such an event are related to traumatic fenestration of the cyst into the subarachnoid cisterns and/or posttraumatic drainage of the cyst into the subdural space [140]. The brain re-expansion is a necessary requisite for the cyst effacement/reduction in seize; indeed, the cyst disappearance is usually found in young patients. As mentioned, this phenomenon could also account for a certain proportion of undiagnosed cases; 6) SAC rupture is a relevant clinical problem affecting the young population but, generally, with a good outcome.

### Management

The previous three sections raise some questions about the strategy and the technical aspects of the management of SACs. The first issue concerns the “preventive” surgery. Indeed, the rupture of SACs has been found to be not exceptional and possibly complicated by a severe clinical picture, especially in young patients. These findings, together with the current possibility to perform mini-invasive and safe cyst fenestration, raise the problem of operating on asymptomatic subjects just to prevent or reduce the risk of rupture. As known, the strategy varies according to the different centers due to the absence of guidelines [141]. However, according to data about complications of surgery, preventive surgery would not be advisable. Actually, the postoperative complication rate of arachnoid cyst surgery is relatively high (range: 8–47%) because of the frequent occurrence of hygroma (6.5–9%), hematoma (5%), and other types of bleeding (3%), neuroendoscopy doing overall better (10% of complications) than microsurgery (20%) [132, 142–153]. These figures indicate that the risk to have a postoperative hygroma/hematoma overlaps or is even higher than the risk to have these collections after a SAC rupture. In addition, the risk of failed surgery must be considered in the decisional process, meaning 1) possible need of re-do surgery for cyst recurrence; 2) possible need of cysto-peritoneal shunt (and subsequent problems related to the shunt dependency). Moreover, also the possibility not to obtain a complete cyst effacement after surgery must be considered. The complete resolution of the radiological picture is reported in about 50% of SACs [4] but the reduction in size of the cyst does not seem to prevent or reduce the risk of rupture, this being not dependent exclusively on the cyst volume [137]. For the same reasons, currently, there is no indication to treat asymptomatic SACs in patients practicing sport activities [130, 135, 154, 155]. These patients have to be correctly informed about the possible risk of rupture and followed-up with dedicated protocols, tailored on the patients’ characteristics and age. However, the participation to sport activities by this subset of patients remains debated because some authors advice a restriction due to the predisposition of athletes to injuries [60].

A second issue is represented by the identification of candidates for surgery after the treatment of the complication of rupture. The complication itself would suggest to treat the associated SAC. Nevertheless, as mentioned before, the complication usually occurs because of an (often misdiagnosed) injury in previously asymptomatic patients and the cyst treatment is not free of complications nor able to eliminate the risk of re-rupture. These arguments as well as the very low risk of re-rupture are against the treatment of ruptured SACs in any case. Indeed, although some authors propose to treat the cyst at the same time or just after the treatment of the complication [115], generally, it is preferred not to operate previously asymptomatic patients [11, 12, 85, 100]. As showed above, we use a personal protocol based on integration of radiological, clinical, and neurophysiological data to identify such candidates. Only children with clear cyst-related symptoms or signs (namely, psychomotor deficits) and/or brain hypoperfusion and/or raised ICP after resolution of the complication undergo the cyst fenestration. Accordingly, 7 out of 16 patients of the personal series needed a cyst fenestration and experienced a good clinical and radiological outcome. The remaining 9 patients did not need any treatment other than the subdural collection evacuation and remained asymptomatic after an almost 10-year-long mean follow-up. Based on this review of the literature, 96 patients (21% of the whole series, 30% of patients with complete information) did not receive any treatment of their SAC. It is worth reminding that information on the treatment is unfortunately missing in a large number of cases (133 patients).

A third, technical issue concerns the management of the complication and the cyst. In spite of the missing information on 86 cases, it can be concluded that a not small number of patients (36 cases, 8%) can be managed conservatively as far as the subdural collection is concerned (Table 2). Sometimes, indeed, the collection (mainly, hygroma) tends to remain relatively thin and not to produce relevant symptoms, so that it is possible to wait for its spontaneous re-adsorption. This phenomenon would explain also the rare disappearance of the cyst after the rupture, due to the drainage into the subarachnoid spaces [30, 134, 140]. Some authors experienced the successful use of acetazolamide for promoting the re-adsorption of the subdural hygroma [39, 131]. On the other hand, other authors were able to obtain the regression of the subdural hematoma by using atorvastatin, thus realizing a conservative, pharmacological management even of this complication (although in a single case) [15]. Burr holes and craniotomy are the main options for the evacuation of the subdural collection. In some instances, the craniotomy is performed to evacuate the subdural hematoma and to fenestrate the cyst microsurgically at the same time [26, 36, 130]. In case of persistent hygroma, the best solution is represented by transient subduro-peritoneal shunt, as demonstrated by the present series and other authors [119]. In these instances, it is mandatory to prevent shunt dependency (over drainage) by using middle or high pressure or adjustable valves.

As far as the treatment of the cyst is concerned, microsurgery resulted the most common option in this review, in spite of the current large use of endoscopy in many centers (the information is missing in 133 cases). This data is explained by the effectiveness of microsurgery and by the fact that several studies here analyzed were carried out before the beginning of the endoscopic era. Another explanation is related to the simultaneous treatment of the subdural collection and the cyst (21% of the whole series, 50% of cases when the cyst was treated). On the other hand, neuroendoscopy and shunt show the same figures. Neuroendoscopy was preferred in the most recent studies [156] while shunt was considered an option mainly in the past or in case of failures of other techniques [119, 157]. Once again, we believe that, in spite of its effectiveness, any attempts should be made to avoid a shunt in this subset of patients. Finally, in 96 cases (21% of the whole series, 30% of cases with complete information), the cyst did not require any treatment and this trend matches the results of our series.

## Conclusions

The “spontaneous” or posttraumatic rupture of SACs is a rare but potentially significant complication followed by a generally good outcome. However, the behavior of the cyst is independent of the outcome of the complication, consequently requiring specific investigations aimed at individuating those lesions interfering with CSF dynamics and/or cerebral blood flow.

## Data Availability

The datasets generated during the current study are available from the corresponding author on reasonable request.
